# Expanding Monomers as Anti-Shrinkage Additives

**DOI:** 10.3390/polym13050806

**Published:** 2021-03-06

**Authors:** Philipp Marx, Frank Wiesbrock

**Affiliations:** 1 Polymer Competence Center Leoben GmbH, Roseggerstrasse 12, 8700 Leoben, Austria; philipp.marx@pccl.at; 2 Chair of Chemistry of Polymeric Materials, Montanuniversitaet Leoben, Otto-Gloeckel-Strasse 2, 8700 Leoben, Austria

**Keywords:** expanding monomer, volumetric shrinkage, volumetric expansion, cationic ring-opening polymerization, radical ring-opening polymerization

## Abstract

Commonly, volumetric shrinkage occurs during polymerizations due to the shortening of the equilibrium Van der Waals distance of two molecules to the length of a (significantly shorter) covalent bond. This volumetric shrinkage can have severe influence on the materials’ properties. One strategy to overcome this volumetric shrinkage is the use of expanding monomers that show volumetric expansion during polymerization reactions. Such monomers exhibit cyclic or even oligocyclic structural motifs with a correspondingly dense atomic packing. During the ring-opening reaction of such monomers, linear structures with atomic packing of lower density are formed, which results in volumetric expansion or at least reduced volumetric shrinkage. This review provides a concise overview of expanding monomers with a focus on the elucidation of structure-property relationships. Preceded by a brief introduction of measuring techniques for the quantification of volumetric changes, the most prominent classes of expanding monomers will be presented and discussed, namely cycloalkanes and cycloalkenes, oxacycles, benzoxazines, as well as thiocyclic compounds. Spiroorthoesters, spiroorthocarbonates, cyclic carbonates, and benzoxazines are particularly highlighted.

## 1. Introduction

**Figure 1:**
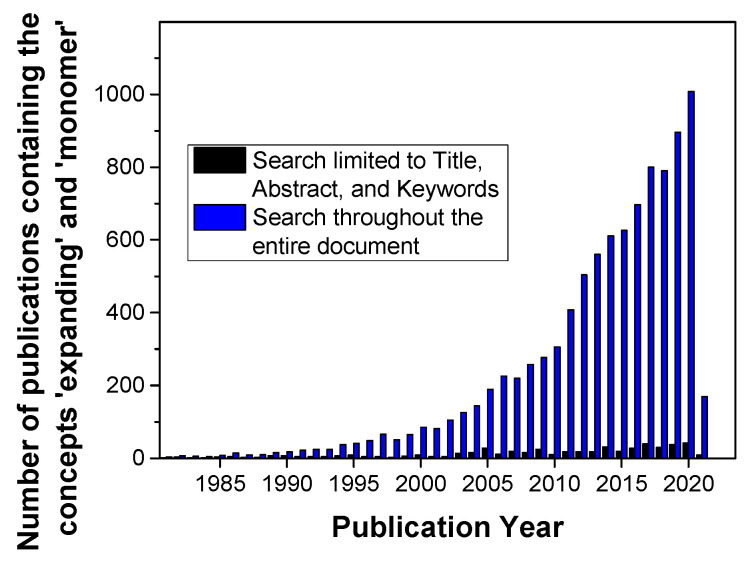
Evolution of the number of publications addressing the concepts of “expanding” and “monomer” during the last three decades. Literature search in the Scopus database on 13 February 2021.

Volumetric aspects of curing reactions in macromolecular science such as polymerizations and polymeranalogous crosslinking reactions have gained continuously increased attention over the recent decades (Figure 1; the blue bars represent the overall activities). Commonly, volumetric shrinkage occurs during curing reactions due to the fact that the Van der Waals distance of two molecules is shortened to the length of a (significantly shorter) covalent bond [1]. This volumetric shrinkage has to be considered for all bonds formed during the curing reaction and can amount to total values higher than 10 vol.% (see the examples reported hereinafter). In final consequence, large-extent volumetric shrinkage induces the formation of voids and micro-cracks in the polymer-based materials as well as delamination of the polymer-based materials from adjacent surfaces [2,3].

Correspondingly, the volumetric shrinkage can have severe influence on the materials’ properties, to mention the deterioration of the optical, mechanical, and insulating properties as prominent examples [4,5,6]. Three main strategies exist to overcome volumetric shrinkage. One of these strategies foresees to apply composite materials with large filler contents: While the fillers are not involved in the curing reaction, volumetric shrinkage only occurs in the share of the total volume occupied by the molecules involved in the curing reaction; the overall shrinkage, hence, is reduced accordingly. The mechanical properties of the composites, however, differ significantly from those of the unfilled polymers and resins [7], which limits the applicability of this strategy. A second strategy foresees the involvement of pre-cured oligomers or even polymers: Due to the fact that a large extent of volumetric shrinkage has already occurred during pre-curing, lower volumetric shrinkage will occur during the (final) curing itself. Nonetheless, both of these strategies only enable to reduce the volumetric shrinkage; the opposite of volumetric expansion cannot be accomplished by these two strategies.

**Figure 2:**
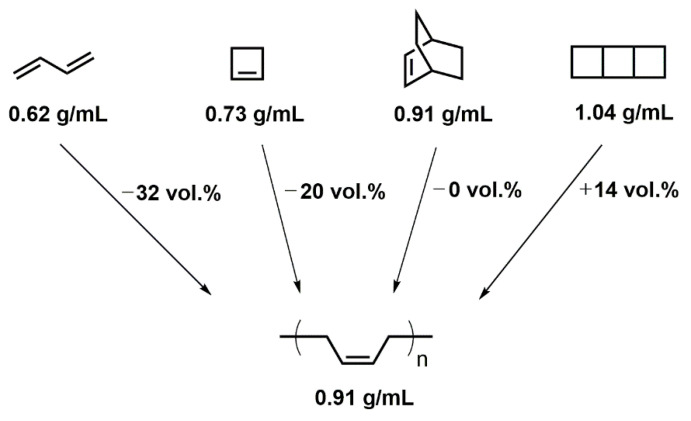
Molecular structure and density of 1,3-butadiene, cyclobutene, bicyclo[2.2.2]oct-2-ene, tricyclo[4.2.0.0^2,5^]octane, and poly(butadiene).

Finally, the third strategy foresees the involvement of so-called expanding monomers; these are monomers that show volumetric expansion during polymerization reactions (Figure 1; the black bars are considered to be indicative of research activities in this field). Only very few monomer classes exhibit this unconventional behavior; these types of monomers exhibit cyclic or even oligocyclic structural motifs. This behavior can be retraced to the dense atomic alignment in small cyclic structures (Figure 2). 

The density of 1,3-butadiene (0.62 g·mol^−1^; no ring present) [8], cyclobutene (0.73 g·mol^−1^; 1 ring present) [9], bicyclo[2.2.2]oct-2-ene (0.91 g·mol^−1^; 2 rings present) [10], and tricyclo[4.2.0.0^2,5^]octane (1.04 g·mol^−1^; 3 rings present) [11] increases alongside with the number of rings present in the molecular structure, indicative of a comparably dense atomic packing in the rings. Theoretical consideration of polymerizations of these “monomers” with the molecular formulae of (C_4_H_6_)_n_ (n = 1, 1, 2, 2) yielding poly(butadiene) with the molecular formula of (C_4_H_6_)_∞_ (density of 0.91 g·mol^−1^) [12] reveals that the polymerizations of 1,3-butadiene and cyclobutene would experience volumetric shrinkage, while the polymerization of bicyclo[2.2.2]oct-2-ene would proceed in volume-neutral fashion and only that of tricyclo[4.2.0.0^2,5^]octane would show volumetric expansion.

Hence, due to ring-opening in the course of the curing reaction, the expanding monomers are likely to show reduced volumetric shrinkage or even volumetric expansion. In the context of reviews of the last decades [13,14,15,16,17], this review aims to provide a concise overview of expanding monomers with a focus on the most recent activities and the elucidation of structure-property relationships. While different methods for the quantification of volumetric expansion exist, which renders it difficult to compare the values reported in the literature, this review is preceded by a brief introduction of measuring techniques for the quantification of volumetric changes. Subsequently, the most prominent classes of expanding monomers will be presented and discussed, namely cycloalkanes and cycloalkenes, oxacycles, benzoxazines, as well as thiocyclic compounds. The applicability of expanding monomers in novel products and materials, with a special focus on bifunctional monomers, which simultaneously exhibit shrinkage (due to, e.g., polymerization or crosslinking) and expansion due to ring-opening reactions, is discussed in a dedicated section.

## 2. Measurement Techniques for Volumetric Shrinkage/Expansion

Various techniques have been developed to measure the volumetric and dimensional changes during crosslinking of thermosetting resins and composites. This chapter gives a short summary of the commonly used techniques, based on volume dilatometry and linear (axial) shrinkage measurements. Potential shortcomings of the individual methods have been summarized at the beginning of this section (Table 1). More detailed descriptions and comparisons of different techniques for the measurement of curing shrinkage can be found in the literature [18,19,20].

### 2.1. Measurement of Volume or Density Changes

**Figure 3:**
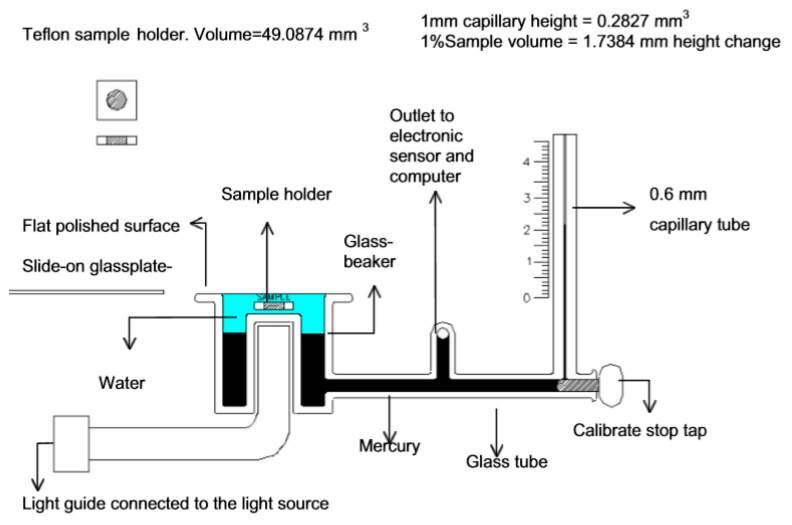
Schematic image of the glass parts of a dilatometer and the Teflon sample holder (top left). Reprinted from reference [23], published first on 4 December 2001. © IOP Publishing. Reproduced with permission. All rights reserved.

In capillary type dilatometers, a sample of the uncured resin or composite is surrounded by a liquid (typically mercury [21] or water [22]), which does not react and is immiscible with the resin (Figure 3). By monitoring the height of the liquid in the capillary, the volumetric changes during polymerization can be monitored [23]. Hence, capillary dilatometers enable the time-resolved monitoring of volumetric changes during the curing reaction. 

**Figure 4:**
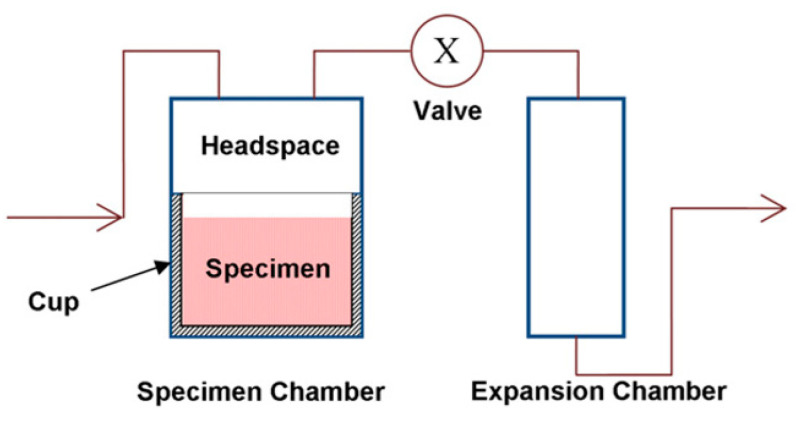
Schematic set-up of a gas pycnometer. Reprinted from reference [26] with permission from Elsevier.

Gas pycnometers enable to quantify volumes by measuring the amount of displaced gas and have been applied for shrinkage measurements of thermosetting resins and composites (Figure 4) [24,25,26]. First, the chamber containing the sample is filled with an inert gas such as helium until a certain pressure is reached. When the valve is opened, the gas expands into the expansion chamber, and the pressure decreases. The volume of the sample V_s_ can be calculated according to Equation (1) [19], in which V_C_ and V_E_ are the volumes of the sample chamber and the expansion chamber, and p_1_ and p_2_ correspond to the pressure before and after expansion of the gas.

(1) $$V_{S}{\;=\;V}_{C}{\;-\;V}_{E}\cdot \frac{p_{2}}{p_{1\;}{-\;p}_{2}}$$

Using the so-called buoyancy method, volumetric changes can be calculated from the density variations of a sample before and after polymerization [18]. The sample is weighed in air and in a liquid of known density such as water or silicon oil. According to Archimedes’ principle, a body immersed in a fluid is buoyed up by a force equivalent to the weight of the fluid displaced by the body. The density ρ of the specimen can be calculated by Equation (2), in which m_l_ and m_air_ correspond to the mass of the sample weighed in the liquid and in air, and ρ_l_ is the density of the liquid:

(2) $$\varrho \;=\;\frac{m_{l}}{m_{\mathrm{air}}{\;-\;m}_{l}}\cdot \varrho_{l}$$

**Figure 5:**
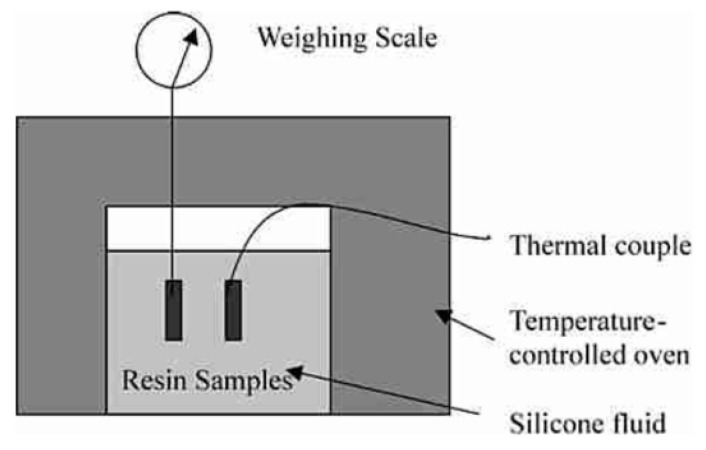
Schematic set-up for the in-situ measurement of curing shrinkage by buoyancy. Reprinted from reference [27] with permission from Elsevier.

Sealing the resin in a thin-walled silicone rubber bag and subsequent suspension in a silicone bath enable the in-situ measurement of the shrinkage of liquid epoxy resins during isothermal curing (Figure 5) [27].

### 2.2. Measurement of Linear Shrinkage

**Figure 6:**
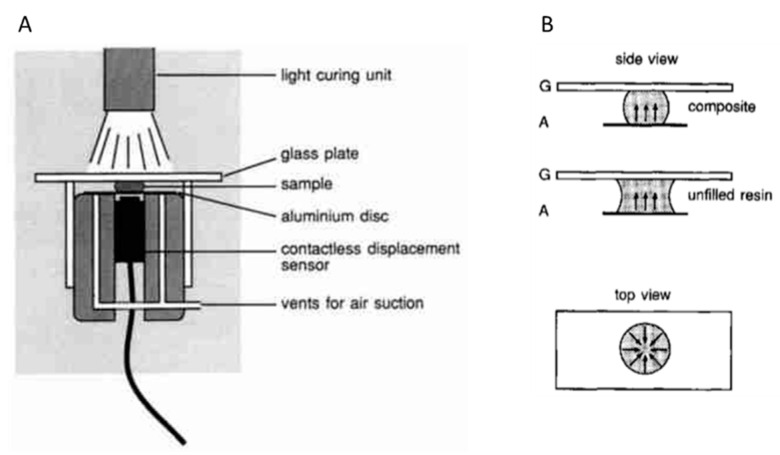
(**A**): Schematic set-up of a linometer. (**B**): Side and top view of the shape of a composite and unfilled resin sample placed between the glass slide and aluminum disk of a linometer. Both pictures (referred to as (**A**,**B**)) constituting this Figure 6 have been reprinted from reference [28] with permission from Elsevier.

A so-called linometer was developed by Gee et al. It enables the measurement of the linear shrinkage during the curing of resins and composites (Figure 6) [28]. In this device, the sample is placed between a glass slide and an aluminum disk. The materials to be investigated can be photocured by irradiation through the glass slide. The linear shrinkage in vertical direction can be monitored during the curing reaction by measuring the displacement of the aluminum disk with a contactless displacement transducer. 

The linear shrinkage LS can be calculated according to Equation (3), in which ΔL is the recorded displacement and L is the height of sample after polymerization:

(3) $$\mathrm{LS}\;(\%)\;=\;\frac{\Delta L}{L\;+\;\Delta L}\cdot 100$$

From the linear shrinkage LS, the volumetric shrinkage VS can be calculated according to Equation (4):

(4) $$\mathrm{VS}\;(\%)\;=\;3\cdot \mathrm{LS}\;-\;0.03\cdot {\left(\mathrm{LS}\right)}^{2}\;+\;0.0001\cdot {\left(\mathrm{LS}\right)}^{3}$$

The observed shrinkage values by Gee et al. were in good agreement with the results of dilatometry measurements that were performed for comparison. 

The bonded-disk method was developed to measure the real-time linear shrinkage of dental composites during curing [29]. A disk-shaped specimen is placed between a rigid glass plate and flexible microscope cover slip. The displacement during the curing reaction by irradiation through the lower glass plate is measured by a linear variable differential transformer (LVDT), and the shrinkage in vertical direction can be calculated from the displacement and height of the specimen after curing (Equation (3)) [30].

The application of lasers for the measurement of linear shrinkage has been used in several approaches referred to as laser interferometer method [31], laser beam scanning, [32] and laser reflection method [33,34]. Compared to mechanical techniques, the vertical displacement of resins and composites caused by shrinkage during the curing reaction can be determined with high resolution in the range of 10 nm to 0.5 μm [19].

Rheometers have been used for the measurement of the linear shrinkage of epoxy resins [35] and acrylate [36] resins in dependence of the reaction time. After the gelation point of the resin, the gap change between the upper and lower plate of the rheometer was recorded. Coupling with near-infrared spectroscopy enabled to correlate the conversion with the volumetric shrinkage during photopolymerizations [36]. Thermomechanical analyzers have been used to quantify the linear polymerization shrinkage by monitoring the height of the resin and composite samples during thermal curing [26,37].

### 2.3. Imaging Methods

Another technique to quantify the volumetric shrinkage of thermally or photocured resins is video-imaging [38,39]. For the measurement, a small drop of the resin (typically 5 to 15 μm) is placed on an adjustable platform, and its contour is recorded by a camera. By dividing the pictures of the drop into thin, horizontal slices with a certain height, the overall volume of the drop can be calculated. The polymerization shrinkage is obtained from the calculated volume of the drop before and after curing.

Digital image correlation DIC is a full-field image analysis method, which enables the measurement of the three-dimensional deformation of a sample [40,41]. By tracking the movement of visible patterns on the sample surface with cameras, the deformation of the sample can be determined during the curing process. A major advantage of DIC is the possibility to measure non-uniform and anisotropic displacements and strain patterns within the sample.

Optical coherence tomography [42] and x-ray micro-computed tomography [43,44] were applied to determine the shrinkage of dental composites during photocuring. Tomographic methods have the advantage that the accuracy of the measurements is not influenced by different shapes of the sample as well as enclosed voids and air bubbles within the material. 

## 3. Expanding Cycloalkanes and Cycloalkenes

### 3.1. Vinylcyclopropanes

**Figure 7:**
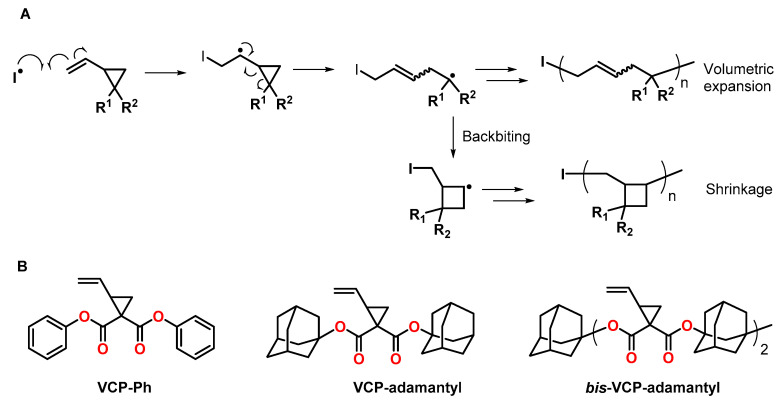
**(A**): Schematic representation of the RROP of vinylcyclopropanes. (**B**): Structures of vinylcyclopropanes that show volumetric expansion during RROPs.

2-Vinylcyclopropanes are monomers that can be subjected to radical ring-opening polymerizations RROPs with low shrinkage compared to linear vinyl monomers [45,46]. The RROP is initiated by the addition of a radical species to the vinylic double bond, followed by ring-opening involving the formation a carbon-carbon double bond and a propagating radical species (Figure 7A). The formation of polymers with ring-opened units provides volumetric expansion during the RROP, while the by-reactions, namely backbiting and the formation of cyclobutane units, cause volumetric shrinkage. In consequence, most vinylcyclopropanes exhibit shrinkage; nonetheless, some examples of monomers with bulky substituents have been reported to undergo volumetric expansion during polymerization (Figure 7B).

During the bulk polymerization of 1,1-bis(phenoxycarbonyl)-2-vinylcyclopropane VCP-Ph (Figure 7B) at 60 °C using azobisisobutyronitrile AIBN as initiator, volumetric expansion of 6.7 vol.% was observed by Ueda et al. [47]. The expansion rate decreased at lower monomer concentrations during solution polymerization as well as with increasing temperature due to the by-reactions of recyclization and the formation of the cyclobutane units.

The synthesis and RROP of 1,1-bis[(1-adamantyloxy)carbonyl]-2-vinylcyclopropane VCP-adamantyl (Figure 7B) with volumetric expansion of 4.5 ± 1.1 vol.% was reported by Shimada et al. [48]. Due to steric hindrance by the adamantyl groups, the unfavored recyclization by-reaction was suppressed, and, even at high temperatures, the ring-opening reaction occurred preferentially. Molecular orbital calculations suggested that the RROP causes a spread of the adamantyl groups, and the free volume of the polymer increases. 

The group of Endo synthesized the bifunctional adamantane-substituted vinylcyclopropane bis-VCP-adamantyl (Figure 7B), which enables the formation of crosslinked networks by RROP [49]. During homopolymerization, volumetric expansion of 6.1 vol.% was observed. By copolymerization of bis-VCP-adamantyl with tricyclodecane dimethanol dimethylacrylate, the shrinkage was reduced from 8.5 (observed in the course of the polymerization of tricyclodecane dimethanol dimethylacrylate) to 3.6 vol.%.

Argawal and coworkers compared the thermally triggered and photo-initiated RROP of 1,1-bis(ethoxycarbonyl)-2-vinylcyclopropane [50]. During photopolymerization, a significantly reduced formation of cyclobutane units and, correspondingly, increased extent of ring-opening reactions was observed in comparison to thermal polymerization. Hence, the shrinkage of 11.2 vol.% during the thermally induced RROP could be reduced to 5.5 vol.% during photopolymerization.

**Figure 8:**
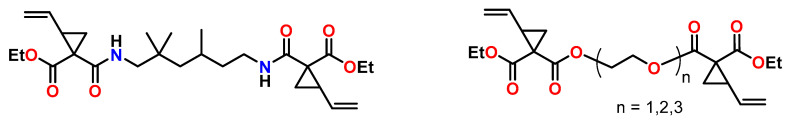
Chemical structure of selected vinylcyclopropanes for potential application in dental composites.

Moszner, Liska and coworkers investigated the photocuring of vinylcyclopropane-based composites as alternative to methacrylate-based dental fillings [51,52,53]. The synthetic focus was directed on various vinylcyclopropane monomers, which contain similar structural units like in commonly used acrylate-based monomers such as urethane dimethacrylate UDMA and tri(ethylene glycol) dimethacrylate TEGDMA (Figure 8). The composites exhibited low volumetric shrinkage in the range of 1.5 to 1.8 vol.%. The shrinkage stress as function of the curing time was measured based on a method described by Watts et al. [54] using a universal testing machine. During polymerization, the vinylcyclopropane monomers exhibited up to 50% lower shrinkage force compared to the corresponding methacrylate-based analogues.

### 3.2. Norbornenes

**Figure 9:**
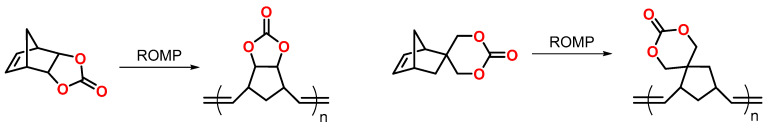
ROMP of norbornenes with five- and six-membered cyclic carbonate groups.

Endo and coworkers reported volumetric expansions in the range of 2.0 to 6.3 vol.% during the ring-opening metathesis polymerization ROMP of norbornenes that contain five- and six-membered cyclic carbonate groups (Figure 9) [55]. Under otherwise identical reaction conditions, the polymerization of unsubstituted norbornenes proceeded with shrinkage of 1.6 vol.%. The unexpected volumetric expansion during the ROMP was explained by dipole–dipole interactions between the cyclic carbonate groups. Prior to the polymerization, these interactions are strong, yielding densely packed monomers. After the ROMP, the dipole–dipole interactions are disturbed due to steric hindrance within the formed polymer structures. In a second step, the linear polymer chains were crosslinked at 60 °C by the cationic ring-opening polymerization CROP of the cyclic carbonate CC units initiated by scandium triflate Sc(OTf)_3_. During crosslinking, volumetric changes ranging from −0.3 to +3.3 vol.% were observed.

In a subsequent study, Endo et al. investigated the influence of the reaction conditions on the expansion rates during the ROMP of norbornenes with five- and six-membered CC groups (Figure 9) [56]. Expansion rates of 1.1 to 3.9 vol.% were observed, depending on the reaction conditions. The polymerizations were performed at 25 °C in dichloromethane CH_2_Cl_2_ using differently substituted Grubbs catalysts. By systematic variation of the initiator and monomer concentration, respectively, the volumetric expansion was found to increase with an increasing monomer concentration, molecular weight, and content of irregular *cis*-configuration of the *exo*-cyclic double bonds in the polymer. The polymerization of structurally similar norbornenes without cyclic carbonate groups proceeded with volumetric shrinkage, indicating that the volumetric expansion during the ROMP exclusively occurs with norbornenes bearing cyclic carbonate moieties.

The volumetric shrinkage during the ROMP of cyclooctene can be tuned by copolymerization with norbornenes that contain cyclic carbonate groups [57]. By variation of the amount of the norbornene derivatives from 0 to 90 mol%, the degree of volumetric changes during the ROMP could be customized in the range of −7.4 to +3.8 vol.%. Simultaneously, the glass-transition temperature T_g_ of the copolymers increased from −60 to 172 °C along with the content of norbornene. The number-average molecular weight M_n_ of the obtained copolymers ranged from 14.5 to 25.8 kDa.

## 4. Expanding Oxacycles

### 4.1. Oxetanes

Oxetanes are a monomer class known to polymerize with lower volumetric shrinkage than other oxacycles such as epoxides, and, hence, have gained attention for applications that require low-shrinking resins [58].

Verstegen et al. investigated the photopolymerization of numerous difunctional oxetanes derived from bisphenol A [59]. Cationic photopolymerization in the presence of triaryl sulfonium salts yielded crosslinked networks with shrinkage lower than 3 vol.%. The oxetane monomer 2,2-di(4-(2-(3-methyloxetane-3-ylmethoxy)propyloxy)phenyl)propane polymerized with pronouncedly low shrinkage of 0.2 vol.%. The T_g_ of the polymer networks ranged from 71 to 116 °C. The polymerization of the oxetanes proceeded with higher reaction rates compared to bisphenol A diglycidyl ether and with lower reaction rates compared to the dimethacrylate diacryl 101. 

The group of Lub reported the synthesis and cationic photopolymerization of liquid crystalline difunctional oxetanes [60]. The effect of the spacer length as well as the structure of the mesogenic and the oxetane group on the liquid crystalline and optical properties was investigated. Photopolymerization at temperatures above 60 °C in the presence of triaryl sulfonium salts as initiator proceeded with almost 100% conversion and low shrinkage of 2 vol.%. Thermally stable films with birefringence values of up to 0.13 were prepared. The synthesized oxetanes polymerized with similar reaction rates like commonly used liquid crystalline dimethacrylates and much faster than liquid crystalline diepoxides.

### 4.2. Spiroorthoesters 

**Figure 10:**
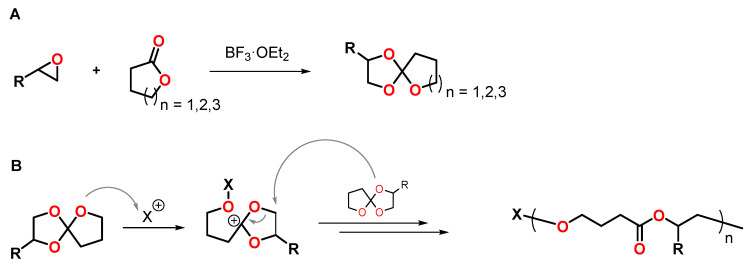
**(A**): Synthesis of SOEs from epoxides and lactones. (**B**): Schematic representation of the cationic double ring-opening polymerization of SOEs.

Spiroorthoesters SOEs are conventionally synthesized by the reaction of lactones with epoxides in the presence of boron trifluoride diethyl etherate BF_3_·OEt_2_ as catalyst (Figure 10A) [61]. After the report of Bailey et al. of the polymerization of spirocyclic compounds upon volumetric expansion in the 1970s, SOEs have gained increased attention as expanding monomers, and numerous studies on the synthesis and polymerization of SOEs were published [13,15]. In the presence of Bronsted and Lewis acids, SOEs polymerize by cationic double-ring opening reaction, yielding poly(ether ester)s (Figure 10B). For the polymerization of seven-membered SOEs at low temperatures, single ring-opening polymerization upon the formation of poly(cyclic orthoester)s was reported [62]. At temperatures above 100 °C, the poly(cyclic orthoester)s undergo isomerization yielding poly(ether ester)s.

The volumetric expansion during the double-ring opening polymerization of SOEs can be referred to the change of bond distances [13]. The shrinkage occurring during the formation of a covalent bond between two monomers is counterbalanced by the double-ring opening, in which two covalent bonds are cleaved upon volumetric expansion.

Mehrkhodavandi et al. prepared a cationic indium-based catalyst for the synthesis of SOEs from lactones and epoxides [63]. The indium complex catalyzes the reaction of 1:1 mixtures of a variety of epoxides and lactones into SOEs, which were obtained as racemic mixtures of diastereomers. At 60 °C in benzene, complete conversion of both educts, the corresponding epoxide and lactone, was observed within 24 h. Competing homopolymerization of the epoxide, which takes place during conventional SOE synthesis with BF_3_·OEt_2_ as catalyst, was completely avoided.

The double ring-opening polyaddition of mono- and multifunctional SOEs and acid chlorides was investigated by Nishida and coworkers [64]. The presence of various tetraphenyl phosphonium halides as catalysts enabled performance of the ring-opening reaction at room temperature with conversions of up to 100%. The polyaddition of a bifunctional SOE and 1,3,5-pentanetricarbonyl trichloride yielded a crosslinked network by curing at 30 °C in the presence of 1 mol% tetraphenylphosphonium bromide. Measurements of the volumetric change during the curing reaction using a dilatometer were carried out over a time period of 10 d and revealed a final shrinkage of 0.21 vol.%.

**Figure 11:**
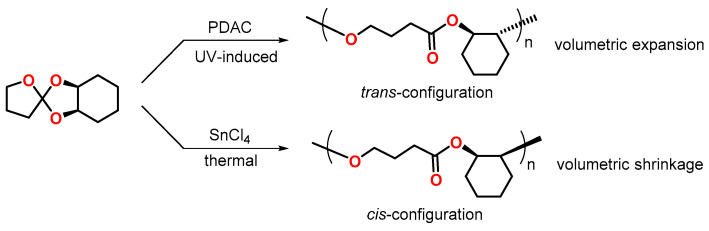
Regiospecific cationic polymerization of *cis*-2,3-tetramethylene-1,4,6-trioxaspiro[4.4]nonane.

Hsu and coworkers demonstrated the regiospecific photopolymerization of *cis*-2,3-tetramethylene-1,4,6-trioxaspiro[4.4]nonane by exposure to UV light (Figure 11) [65]. The photopolymerization, initiated by a sterically hindered 9-phenyl-9,10-dihydroanthracen-10-ylium cation PDAC, yielded poly(*trans*-2-oxycyclohexyl butanoate) with a volumetric expansion of 2.1 vol.%. In contrast, during the thermally initiated polymerization with tin(IV)chloride SnCl_4_ as initiator, the corresponding *cis*-form of the polymer was formed with volumetric shrinkage of 2.5 vol.%. According to density-functional theory DFT calculations, the *trans*-form of the poly(ether ester) shows a volumetrically extended structure, while the *cis*-form exhibits a more compact and coiled conformation, which explains the different volume changes during polymerization. In a subsequent study [66], volumetric expansion rates of 1.53 and 5.35 vol.% were observed during regiospecific photo-polymerization of the SOEs *cis*-2,3-tetramethylene1,4,6-trioxaspiro[4.5]decane and *cis*-2,3-tetramethylene-1,4,6-trioxaspiro[4.6]undecane. 

Cadiz and coworkers compared the cationic copolymerization of 2-phenoxymethyl-1,4,6-trioxaspiro[4.4]nonane and bisphenol A diglycidylether DGEBA with lanthanide triflate La(OTf)_3_ and ytterbium triflate Yb(OTf)_3_ as initiators under microwave-assisted and conventionally heated conditions [67]. Microwave-assisted irradiation was argued to accelerate the reaction rate by a factor of ten. Fourier-transformed infrared FT-IR measurements of the curing reaction revealed a higher conversion of the SOE units under microwave irradiation. This resulted in lower shrinkage in the range of −1.1 to −1.6 vol.% for the microwave-assisted polymerization, compared to −2.2 to −2.6 vol.% for conventional thermal curing. The T_g_ of the obtained networks was in the range of 77 to 80 °C.

Ramis and coworkers investigated the cationic copolymerization of DGEBA and 4-phenyl-γ-butyrolactone in the presence of Yb(OTf)_3_ as thermal initiator and triarylsulfonium hexafluoroantimonate as photoinitiator, respectively [68]. Monitoring of the curing reaction by FT-IR measurements revealed the formation of intermediate SOE compounds and subsequent double-ring opening of the SOE moieties by homopolymerization and copolymerization with epoxy groups. The volumetric shrinkage before and after gelation was determined by thermomechanical analyses TMAs and density measurements. The overall shrinkage slightly increased with the amount of lactone, due to higher conversions. Nevertheless, after gelation, a shrinkage reduction from 0.91 vol.% (neat DGEBA) to 0.21 vol.% (ratio DGEBA:lactone = 1:1) was observed upon the addition of 4-phenyl-γ-butyrolactone. Due to the higher crosslinking density, the photocured networks exhibited higher T_g_s and thermal stabilities compared to thermally cured materials.

**Figure 12:**
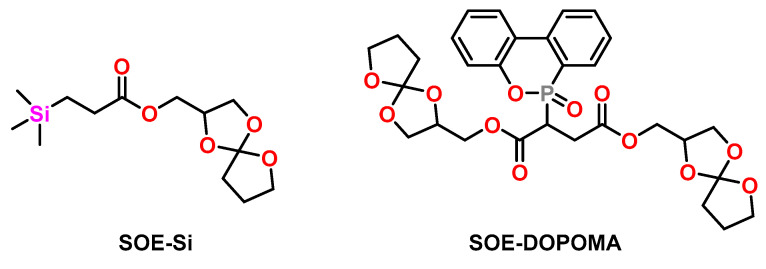
Chemical structure of silicon- and phosphorus-containing SOEs.

Canadell et al. investigated the effect of silicon- [69] and phosphorous-containing SOEs [70] on the shrinkage and flame retardancy of epoxy resins (Figure 12). DGEBA was cured with Yb(OTf)_3_ as cationic initiator at temperatures in the range from 140 to 180 °C with the corresponding SOE in a molar ratio of 2:1. While pure DGEBA exhibited shrinkage of 3 vol.%, the formulations DGEBA/SOE-Si and DGEBA/SOE-DOPOMA (Figure 12) showed volumetric expansion of +0.9 vol.%. The homopolymerization of SOE-Si and SOE-DOPOMA proceeded with volumetric expansion in the range of +3 vol.%. The copolymers containing repetition units of SOE-Si and SOE-DOPOMA showed lower thermal stability and lower T_g_s compared to the DGEBA homopolymer. The limiting oxygen values increased upon the addition of SOE-Si and SOE-DOPOMA. In another study, reduced shrinkage in the range of 0.5 to 0.9 vol.% as well as improved flame retardancy was observed in copolymers of SOE-Si, DGEBA, and phosphorous-containing diglycidyl compounds [71].

The groups of Sangermano and Wiesbrock employed SOEs bearing allyl groups as volume-controlling additive in photocurable thiol-ene resins composed of DGEBA and trifunctional thiol crosslinkers [72]. The volumetric expansion during crosslinking could be tailored in the range of −3.07 to +1.70 vol.%, alongside with an increase of the SOE content from 0 to 30 wt.%. Network formation was accomplished according to a visible-light induced dual-cure mechanism, comprising the radical-mediated thiol-ene reaction and cationic double ring-opening reaction of the SOE groups. 

In a subsequent study, the authors reported the 3D-printing of thiol-ene resins with SOEs as anti-shrinkage additives by digital light-processing [73]. Formulations of tri(ethylene glycol) divinyl ether, a tetrafunctional thiol crosslinker, and 50 wt.% of 2-((allyloxy)methy)-1,4,6-trioxospiro[4.4]nonane were 3D-printed with resolutions of 50 μm, while the shrinkage was reduced by 39 vol.% compared to the resin free of the SOE. The cured networks containing SOEs showed permittivities as high as 10^4^, qualifying the materials as high-κ dielectrics. 

Kawakami and coworkers synthesized siloxane-containing bifunctional SOEs and epoxides via the hydrosilylation of 2-allyloxymethyl-1,4,6-trioxaspiro[4.6]undecane and allyl glycidylether [74]. Mixtures of the SOEs and epoxides with trimethylolpropane triacrylate were used to prepare holographic gratings by irradiation with laser light (λ = 532 nm). The SOE-based holographic gratings showed diffraction efficiency of 66% for the siloxane-containing SOEs and 36% for the aliphatic SOEs. The volumetric shrinkage of the SOE-based formulations (6.8 vol.%) was lower than that of the epoxy-based formulations (11.8 vol.%).

The application of SOEs bearing *exo*-methylene groups as anti-shrinkage additive for dental resins was investigated by Hang et al. [75]. Acrylic resins based on bisphenol S glycidyl methacrylate and 30 wt.% of the SOE were cured by visible light using a three-component photoinitiator system. By addition of the SOE, the curing shrinkage was reduced from 2.92 to 1.34 vol.%. Due to reduced shrinkage stress, the cured polymer networks containing the SOE showed increased compressive strength, Vickers hardness and tensile bond strength.

The copolymerization of the SOE 2-phenoxymethyl-1,4,6-trioxa-spiro[4.6]undecane PSOE with 3-ethyl-3-phenoxymethyl oxetane EPOX was investigated by Nagasawa et al. [76]. PSOE and EPOX were mixed in different molar ratios and copolymerized at 120 °C, initiated by a benzyl sulfonium salt. Both monomers showed nearly complete conversion, while the molecular weight of the copolymers increased along with the amount of EPOX. By variation of the monomer ratio PSOE:EPOX from 0:100 to 100:0, volumetric changes in the range from −3.88 to +3.21 vol.% were observed in the course of the polymerization. IR thermography revealed that the addition of PSOE suppressed temperature increases during polymerization, which would lead to thermal runaway and thermal shrinkage. Polymer networks were obtained from the curing reaction of the bifunctional oxetane 1,4-bis(3-ethyloxetanylmethoxy)benzene with PSOE in the ratio of 80:20 with volumetric expansion of 3.7 vol.% [77].

**Figure 13:**
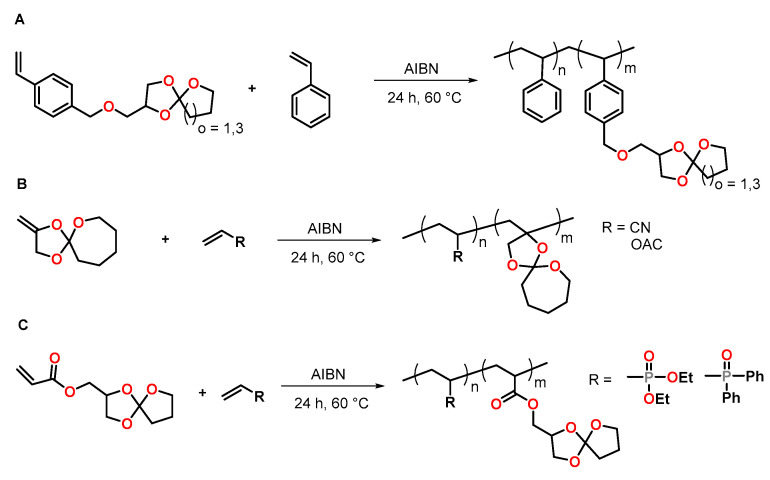
Structural representation of polymers with crosslinkable SOE groups in the sidechain. (**A**): Copolymers of styrene and five/seven-membered SOEs. (**B**): Copolymers of acrylonitrile or vinyl acetate with five/seven-membered SOEs bearing unsaturated C=C double bonds. (**C**): Copolymers of phosphorous-containing vinyl monomers and SOEs with acrylate groups.

Novel styrene monomers bearing five- and seven-membered SOE groups (SOE5 and SOE7) were synthesized from 4-vinylbenzyl glycidylether and γ-butyrolactone or ε-caprolactone, respectively, by Endo and coworkers (Figure 13A) [78]. The radical polymerization of these monomers as well as the crosslinking of the obtained polymers was investigated. Radical copolymerization of the SOEs with styrene was performed with AIBN as initiator at 60 °C in methyl ethyl ketone MEK; the corresponding statistic copolymers were obtained with yields between 44 and 74%. The copolymers were crosslinked by cationic double ring-opening of the SOE units in the sidechains at 120 °C in the presence of a sulfonium antimonite as thermally latent cationic initiator. The crosslinking reaction occurred with low volumetric shrinkage. During the crosslinking of the homopolymer poly(SOE7) and the copolymer polystyrene_0_._32_-*co*-poly(SOE7)_0_._68_, volumetric expansions of 2.78 and 0.42 vol.% were achieved, respectively.

Endo et al. investigated the radical copolymerization of a SOE bearing an *exo*-methylene group with acrylonitrile and vinylacetate (Figure 13B) [79]. The radical copolymerization was performed at 60 °C with AIBN as initiator for 24 h. No radical ring-opening polymerization of the SOE was observed, and statistical copolymers with SOE moieties in the sidechain were obtained. During crosslinking with BF_3_·OEt_2_ as cationic initiator at 120 °C for 24 h, all copolymers showed volumetric expansion in the range from 0 to +1 vol.%, regardless of the copolymer composition. 

Cadiz et al. reported the homopolymerization of SOEs bearing acrylate groups and the copolymerization with phosphorous-containing vinyl monomers (Figure 13C) [80]. Upon free-radical polymerization, copolymers with different phosphor concentrations and crosslinkable SOE units in the sidechain were obtained. By crosslinking with Y(OTf)_3_ as cationic initiator at 80 °C, networks with a T_g_ in the range of −14 to +12 °C were obtained. All copolymers showed volumetric expansion of +1.7 to +2.6 vol.% during crosslinking. The incorporation of 3 and 6 wt.% phosphorous in the copolymers increased the limiting oxygen values and the flame retardancy of the materials. 

Radical homopolymerization of the acrylic SOE monomer yielded a polyacrylate (M_w_ of 25.0 kDa) with SOE side-groups. Crosslinking with DGEBA and various phosphorous-containing epoxy components proceeded with low shrinkage in the range of −0.2 to −0.7 vol.% and yielded networks with a T_g_ above 100 °C and increased limiting oxygen values [81].

### 4.3. Spiroorthocarbonates

**Figure 14:**
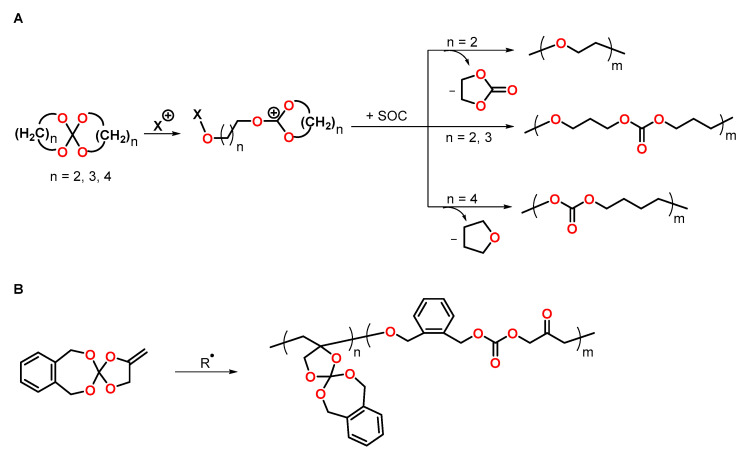
(**A**): Schematic representation of the cationic ring-opening polymerization of SOCs. (**B**): Schematic representation of the radical polymerization of SOCs bearing *exo*-methylene groups.

Since the first report by Bailey and coworkers of the spiroorthocarbonate SOC polymerization upon volumetric expansion in 1972, the synthesis and application of SOCs as expanding monomers to control the shrinkage during conventional polymerizations has gained increased attention [13,17]. In the presence of acids, six-membered SOCs undergo cationic double ring-opening polymerization upon volumetric expansion, yielding poly(ether carbonate)s (Figure 14A). For SOCs that contain five- or seven-membered rings, the formation of polyethers and polycarbonates, respectively, has been reported [82]. Due to the accompanying elimination of propylene carbonate and tetrahydrofurane THF as by-products, the application of five- and seven-membered SOCs as expanding monomers is limited. SOCs that bear *exo*-methylene groups can undergo radical-initiated vinyl polymerization and radical-initiated double ring-opening polymerization (Figure 14B) [83,84]. The degree of ring-opening depends on the monomer structure and the stability of the radical intermediates formed. The volumetric expansion upon polymerization increases alongside with the degree of double ring-opening.

**Figure 15:**
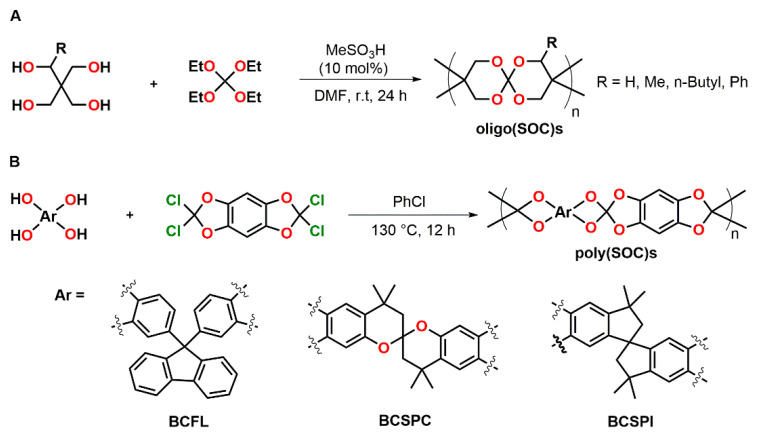
(**A**): Synthesis and structural formula of oligo(SOC)s. (**B**): Synthesis and structural formula of poly(SOC)s.

The synthesis of oligo(spiroorthocarbonate)s by polycondensation of pentaerythritol derivatives and tetraethyl orthocarbonate was reported by Endo and coworkers (Figure 15A) [85]. Oligomers with a M_n_ in the range of 1.1 to 1.3 kDa and dispersity indices in the range of Đ = 1.66 to 1.96 were obtained. The solubility is strongly dependent on the substituent R (Figure 15A). While the oligomeric SOC with R = H was completely insoluble, the introduction of methyl, *n*-butyl and phenyl groups provided solubility in common organic solvents.

Moritsugu et al. synthesized novel poly(SOC)s from several bis-catechols containing fluorine BCFL (yielding SOC-FL), spirobischromane BCSPC (yielding SOC-SPC), and spirobisindane BCSPI (yielding SOC-SPI) structures (Figure 15B) [86]. Polymers were obtained as white solids with weight-average molecular weights M_w_ in the range of 10.8 to 35.8 kDa. From the soluble polymers poly(SOC-FL) and poly(SOC-SPC), transparent films with 95% light transmission in the visible region were prepared by spin-coating. In the temperature range from 50 to 300 °C, no T_g_ was detected. The polymers showed high thermal stability. Data on the ring-opening reaction of the SOC groups and expansion measurements were not reported.

The group of Endo investigated the cationic copolymerization of oligomeric SOCs (Figure 15A) with DGEBA [87]. The copolymerization was performed at 180 °C for 1 h in the presence of sulfonium salts as cationic initiators. The methyl-substituted SOC showed higher shrinkage reduction compared to other SOCs. While pure DGEBA showed shrinkage of 3.1 vol.%, mixtures with 1 and 10 mol% of the methyl-substituted oligo-SOC exhibited shrinkage of only 1.2 and expansion of +0.1 vol.%, respectively. The copolymer networks containing oligo(SOC)s showed a similar temperature stability like the DGEBA homopolymer.

**Figure 16:**
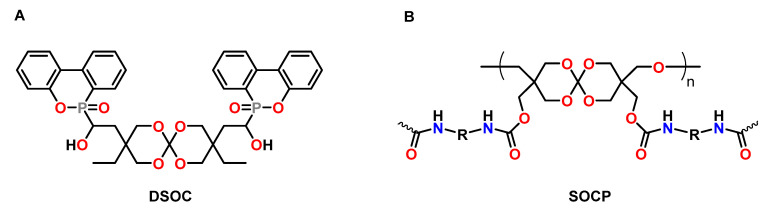
(**A**): Chemical structures of the phosphorous-containing SOC DSOC. (**B**): Chemical structure of the SOC-based prepolymer SOCP.

Xu et al. synthesized novel phosphorous-containing SOCs DSOCs as anti-shrinkage additive and flame-retardant for epoxy resins (Figure 16A) [88]. The DSOC was copolymerized with the DGEBA-based epoxy resin E51 at 150 °C in the presence of boron trifluoride triethylamine BF_3_·NEt_3_ as catalyst. During crosslinking, volumetric changes in the range from −1.49 to +1.43 vol.% were observed if the content of DSOC was increased from 0 to 20 wt.%. The limiting oxygen index LOI values increased from 19.9% for the epoxy resin to 30.9% for the resin containing 20 wt.% DSOC.

The prepolymer SOCP was synthesized from an oligomeric SOC bearing hydroxy functions and toluene diisocyanate by Changsong and coworkers (Figure 16B) [89]. During copolymerization of the DGEBA-based epoxy resin E51 with 20 wt.% of SOCP at 190 °C in the presence of BF_3_·NEt_3_, volumetric expansion of 1.1 vol.% was observed. The polymer networks containing SOCP showed increased adhesive strength, impact strength, and flexural strength compared to the pure epoxy resin.

Ortiz et al. reported the shrinkage reduction of cycloaliphatic epoxy resins by copolymerization with sterically hindered SOCs [90]. A dicyclododecanyl-substituted SOC was synthesized with an overall yield of 37% in five steps starting from cyclodecanone. The SOC was copolymerized with 3,4-epoxycyclohexylmethy-3′,4′-epoxycyclohexane carboxylate by cationic photopolymerization. Upon addition of 4 and 8 mol% of the SOC, volumetric expansions in the range from 0.68 and 3.39 vol.% were achieved. The T_g_ of the cured polymer networks slightly decreased from 154 °C of the pure epoxy resin to 151 and 140 °C of the materials containing 4 and 8 mol% SOC. 

The cationic photopolymerization of DGEBA with a SOC bearing two fluorenyl groups SOC-Fl as anti-shrinkage additive was investigated by Sangermano et al. [91]. The shrinkage during the curing reaction of pure DGEBA was 4.77 vol.%. Upon addition of 2.5 and 5.0 mol% of SOC-Fl, the shrinkage was reduced to 2.42 and 1.94 vol.%, respectively. For the formulation containing 10 mol% of SOC-Fl, expansion of 0.19 vol.% was observed. All networks showed high gel contents in the range of 98 to 100%, and T_g_s in the range from 157 to 181 °C. The conversion of epoxy groups increased from 70 to 80% alongside with the concentration of SOC-Fl.

Cheng and coworkers reported the preparation of an UV-curable resist for nanoimprint lithography [92]. A bifunctional cycloaliphatic epoxy resin was formulated with a liquid SOC, namely 3,9-diethyl-3,9-bis(allyloxy)-1,5,7,11-tetraoxaspiro undecane and a photoacid generator in different ratios. Upon addition of the SOC, the shrinkage decreased from 7.86 vol.% of the pure epoxy resin to 1.86 vol.% for the formulation containing 50 wt.% of the SOC. In addition, the demolding force during the nanoimprint process was reduced by 69% upon addition of 50 wt.% of the SOC. All formulations were patterned by UV nanoimprinting with a maximum resolution of approximately 60 nm.

**Figure 17:**
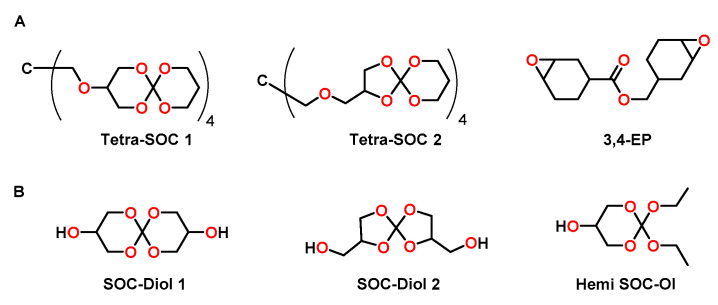
(**A**): Chemical structure of tetrafunctional SOCs and the cycloaliphatic epoxy monomer 3,4-EP. (**B**): Chemical structure of hydroxyl-modified SOC-Diol 1 and SOC-Diol 2 as well as Hemi SOC-Ol.

The groups of Ortiz and Sangermano used tetrafunctional SOCs Tetra-SOCs as anti-shrinkage additive for the photopolymerization of the bifunctional cycloaliphatic epoxide 3,4-EP (Figure 17A) [93]. The Tetra-SOCs were synthesized in three steps starting from tetraethyl orthocarbonate and glycerol with an overall yield of 15%. Since the two obtained isomers were difficult to separate, a mixture (ratio Tetra-SOC 1:Tetra-SOC 2 = 65:35) was used for the cationic copolymerization with 3,4-EP (Figure 17A) in the presence of a iodonium salt as photoacid generator. The epoxy resin without additive showed shrinkage of 5.8 vol.%, which was not significantly reduced by the addition of 2.5 mol% of Tetra-SOC (shrinkage of 5.5 vol.%). On the contrary, addition of 5.0 and 7.0 mol% of Tetra-SOC resulted in nearly volume-neutral curing (+0.26 vol.%) and expansion of +2.2 vol.%, respectively. For the epoxy homopolymer, a T_g_ of 153 °C was observed, which decreased to 125 °C upon the addition of 7 mol% of Tetra-SOC.

Ortiz, Sangermano and coworkers reported the cationic photopolymerization of 3,4-EP with different amounts of hydroxyl-modified SOCs as anti-shrinkage additive (Figure 17B) [94]. By the reaction of tetraethyl orthocarbonate and glycerol, SOC-Diol 1 and SOC-Diol 2 were obtained as hardly separable isomeric mixture in the ratio of 3:2 with a yield of 50%. Hemi SOC-Ol was obtained as by-product in a yield of 25%. Upon addition of a mixture of SOC-Diol 1 and SOC-Diol 2, the conversion of epoxy groups increased from 40% (pure epoxy resin) to 78% (formulation with 20 mol% of the SOC-Diol). The higher conversions were explained by the flexibilization of the polymer networks due to the formation of poly(ether carbonate) chains during the cationic ring-opening of SOC groups and the resulting delay of gelation. Correspondingly, due to the higher conversion, the T_g_ increased from 105 °C for the pure epoxy resin to 145 °C for the formulation containing 5 mol% of the SOC-Diol. However, in networks with further increased concentrations of the SOC-Diol (10 and 20 mol%), the T_g_ decreased to 96 and 81 °C, respectively, due to the formation of flexible poly(ether carbonate) chains. Compared to the pure epoxy resin (shrinkage of 3.97 vol.%), the formulations containing 5, 10 and 20 mol% of the SOC showed reduced shrinkage of 2.83, 2.47 and 2.17 vol.%. 

In another study, the same authors investigated the photopolymerization of DGEBA with a mixture of SOC-Diol 1 and SOC-Diol 2 as well as Hemi SOC-Ol (Figure 17B) [95]. The shrinkage of 8.0 vol.% in the case of pure DGEBA was reduced to 1.8 vol.% for formulations containing 30 wt.% of Hemi SOC-Ol as anti-shrinkage additive. SOC-Diol was found to be more effective, inducing a volumetric expansion of 1.1 vol.% during crosslinking if it was added in the same amount. Gel contents above 95% confirmed the formation of crosslinked networks. The T_g_ of the pure, cured epoxy resin of 145 °C decreases to 128 and 95 °C for the networks containing 30 wt.% SOC-Diol and Hemi SOC-Ol, respectively. While the elastic modulus decreased upon the addition of Hemi SOC-Ol, no significant changes were observed for the SOC-Diol based networks.

**Figure 18:**
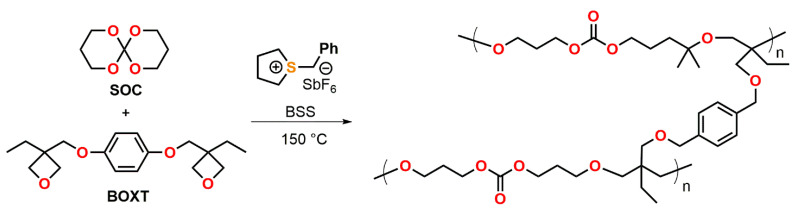
Thermally initiated, cationic copolymerization of a SOC with the bifunctional oxetane BOXT.

Endo et al. described the thermally initiated, cationic copolymerization of a SOC with the bifunctional oxetane BOXT (Figure 18) [96]. By curing formulations containing the bifunctional oxetane and a varying content of the SOC (0, 25, 32, 50, 75 mol%) at 150 °C for 2 h, insoluble polymer networks were obtained with yields in the range from 88 to 96%. While the formulation containing 32 mol% of the SOC were cured without the occurrence of any volumetric changes, the formulations with 50 and 75 mol% of the SOC showed expansion rates of +3.1 and +4.4 vol.% during curing. The T_g_ of the copolymer networks ranged from 86 to −21 °C and was found to decrease linearly with increasing amount of SOC. The copolymerization of the monofunctional monomer 3-ethyl-3-phenoxymethyl oxetane with SOC yielded linear, soluble polymers with M_n_ in the range of 5.6 to 12.0 kDa. The formation of copolymers containing both, repetition units of poly(SOC) as well as poly(oxetane), was proven by proton nuclear magnetic resonance ^1^H-NMR spectroscopy. By increasing the amount of the SOC from 0 to 75 mol%, volumetric changes in the range of −4.5 to +8.6 vol.% were observed.

The cationic UV-induced curing of a bifunctional oxetane resin with an oxetane-functionalized hemi-SOC, namely 7,7-diethoxy-2,6,8-trioxaspiro[3.5]nonane, as anti-shrinkage additive was investigated by the groups of Sangermano and Ortiz [97]. While the pure oxetane resin showed shrinkage of 4.21 vol.%, formulations containing 50 mol% of the hemi-SOC showed slight expansion of +0.95 vol.% upon curing. Both networks showed high gel contents of 97%. The T_g_ of the polymer network decreased from 115 to 70 °C upon the addition of the hemi-SOC. 

**Figure 19:**
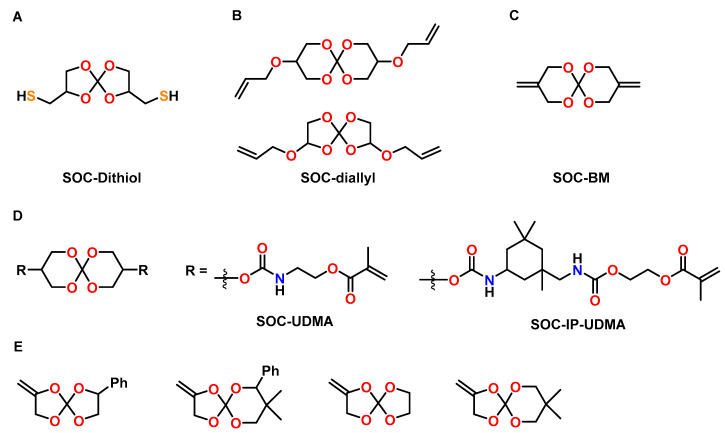
(**A**–**E**): Chemical structure of SOC monomers used as anti-shrinkage additives in acrylic-based dental resins and composites.

The effect of a SOC bearing two thiol groups (SOC-Dithiol) on the mechanical properties and shrinkage of dental resins was investigated by Ortiz et al. (Figure 19A) [98]. The SOC-Dithiol was added to a conventional acrylic resin composed of bisphenol A glyceroate dimethacrylate (bis-GMA), urethane dimethacrylate (UDMA), and tri(ethylene glycol) dimethacrylate (TEGDMA) with concentrations in the range of 5 to 30 mol%. Curing of the resin in the course of both, the radical-mediated thiol-ene reaction and the methacrylate homopolymerization, was initiated by LED light in the presence of a radical initiator; for the cationic ring-opening reaction of the SOC units, also a photo-acid generator was added. The addition of 30 mol% of the SOC-Dithiol reduced the shrinkage by 59% (from 5.37 to 2.21 vol.%) compared to the pure resin without the SOC. The conversion of methacrylate groups increased with the amount of the SOC-Dithiol, which results in higher crosslinking densities and increased elastic modulus. The T_g_ of all materials was in the range from 75 to 90 °C.

A mixture of SOCs with five and six-membered rings bearing two allyl groups (SOC-diallyl) was used to reduce the shrinkage of acrylic dental resins (ratio bis-GMA:UDMA:TEGDMA = 50:30:20) by Ortiz, Gonzales and coworkers (Figure 19B) [99]. The SOC-diallyl was incorporated into the acrylic resin by radical polymerization of the carbon double bonds, while cationic polymerization of SOC groups yielded additional crosslinking by the formation of poly(ether carbonate) chains. The resin without additive showed shrinkage of 5.98 vol.%, which was reduced by 53% upon addition of 20 mol% of the SOC-diallyl. The conversion of the acrylic carbon double bonds increased with the amount of the SOC-diallyl, resulting in higher crosslinking density. Correspondingly, the T_g_s, flexural strength, and compressive strength of the cured networks were found to increase by the addition of the SOC-diallyl.

Dental composites containing bismethylene modified SOCs (SOC-BM), bisphenol S glyceroate dimethylacrylate, and TEGDMA as resin matrix, as well as 70 wt.% of SiO_2_ were prepared by the group of Wang (Figure 19C) [100]. The composites without the SOC exhibited shrinkage of 4.5 vol.% and contraction stress of 3.8 MPa upon curing. In comparison, the composites containing 30 wt.% of the SOC-BM showed reduced shrinkage of 1.25 vol.% and reduced contraction stress of 1.9 MPa. In addition, the compressive strength increased from 200 to 240 MPa upon the addition of the SOC-BM. The cured composites containing SOC-BM showed lower wettability compared to the SOC-free composites. 

Spiroorthocarbonates modified with dimethacrylate urethanes UDMA-SOC and isophorone urethanes IP-UDMA-SOC were synthesized as anti-shrinkage matrices for dental composites by Duarte et al. (Figure 19D) [101]. Formulations of an acrylic resin containing bis-GMA, UDMA and TEGMA as well as different amounts of the SOCs were investigated. The shrinkage stress was reduced by 53% and 47% if bis-GMA was replaced by UDMS-SOC and IP-UDMA-SOC, respectively. The cured polymer networks with IP-UDMA-SOC showed higher flexural strength and elastic modulus compared to pure bis-GEMA-based resins. The T_g_ values of the polymer networks were in the range of 80 to 100 °C.

Kim and coworkers investigated various formulations of alkoxylated bis-GMA derivatives and SOCs containing *exo*-cyclic carbon double bonds for the production of dental composites with reduced shrinkage (Figure 19E) [102]. In the case of thermally induced homopolymerizations of the SOC monomers with AIBN as initiator at 100 °C, volumetric expansions in the range from 2.7 to 4.0 vol.% were observed; photopolymerization in the presence of camphorquinone, on the other hand, was found not to occur. However, during the photocuring of formulations composed of bis-GMA and SOCs, the formation of carbonate groups was detected, indicative of the double ring-opening polymerization of the SOC moieties. A composite composed of a conventional resin matrix (ratio bis-GMA:TEGDMA = 70:30) and 70 wt.% of silica filler showed shrinkage of 2.5 vol.% during the curing reaction. In comparison, the composites containing alkoxylated bis-GMA derivatives and 10 or 20 wt.% of the SOC showed reduced shrinkage in the range of 0.65 to 1.15 vol.%.

### 4.4. Cyclic Carbonates

The polymerizations of six-membered cyclic carbonates CCs can be initiated by either cationic or anionic initiators, yielding polycarbonates almost quantitatively [14]. As expanding monomers, CCs have gained increased attention since the early 1990s, and various six- and seven-membered CCs were found to undergo volumetric expansion in the range of 1.1 to 7.7 vol.% during cationic or anionic homopolymerization [103]. The observed volumetric expansions during polymerization can be explained by a strong difference between some physical properties of cyclic and acyclic carbonates (Table 2) [13]. Despite similar molecular weights, cyclic propylene carbonate shows a higher boiling point, density and dipole moment than acyclic diethyl carbonate. Tanaka et al. proposed that the volume expansion during polymerization is a direct consequence of the changes of the dipole moments before and after polymerization [103]. While CC monomers are densely packed and show strong interactions due to the high dipole moments, these intermolecular interactions are significantly reduced in the corresponding polymers derived from acyclic carbonate units. 

The dipole moment of numerous six-membered CCs that undergo volumetric expansion during polymerization and their acyclic counterparts was determined by semi-empirical molecular orbital calculations [103]. The calculations revealed dipole moments of approximately 5.5 Debye for the CCs, and dipole moments in the range from 0.59 to 1.05 Debye for their acyclic analogues. The volumetric expansion during polymerization was found to increase with the difference of the dipole moments of the monomer and the corresponding polymer.

**Figure 20:**
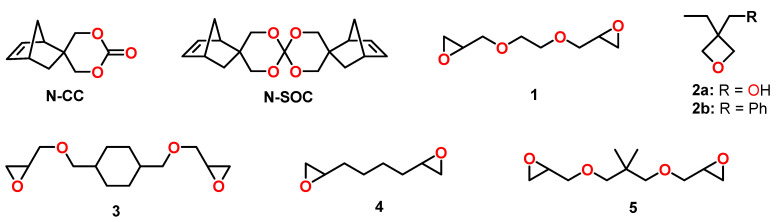
Chemical structure of cyclic ether monomers used for copolymerization with the cyclic carbonate N-CC and the spiroorthocarbonate N-SOC.

The cationic copolymerization behavior of various cyclic ether monomers with norbornene modified cyclic carbonate N-CC was investigated by the group of Endo (Figure 20) [104]. Polymerizations were performed in bulk at 120 °C in the presence of the thermal latent cationic initiator 2-indanyl phenyl methyl sulfonium hexafluoroantimonate. While the cationic homopolymerization of the epoxy monomer 1 (Figure 20) resulted in shrinkage of 24 vol.%, the addition of 5, 10, 20, and 50 mol% of N-CC resulted in volume changes ranging from −19 to +2.5 vol.% during copolymerization. Due to the poor solubility of N-SOC in formulations containing the epoxy monomer, the shrinkage reduction and the yields of copolymer networks were lower compared to the formulations containing N-CC. For the copolymerization of N-CC with the epoxides 3, 4, and 5 (Figure 20), no expansion was observed, but the shrinkage was reduced significantly. From the copolymerization of N-CC with the monofunctional oxetane 2a, linear copolymers with M_n_s in the range from 5.8 to 6.9 kDa were obtained; the formulation containing 30 mol% N-CC showed expansion of 0.4 vol.% during polymerization.

**Figure 21:**
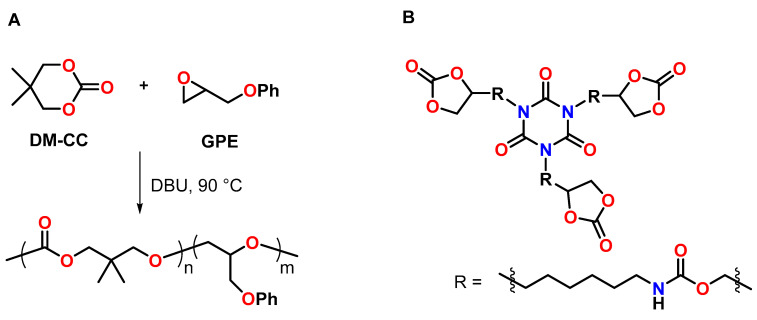
(**A**): Copolymerization of the cyclic carbonate DM-CC with phenyl glycidylether GPE. (**B**): Chemical structure of trifunctional cyclic carbonates.

The anionic copolymerization of the cyclic carbonate DM-CC with phenyl glycidylether GPE in the presence of 4 mol% 1,8-diazabicyclo[5.4.0]undec-7-ene DBU as anionic initiator was reported by Morikawa et al. (Figure 21A) [105]. The homopolymerization of GPE at 90 °C proceeded slowly; after 90 h, the conversion of the epoxy groups amounted to only 39%. The addition of DM-CC accelerated the polymerization rate of GPE and suppressed chain-transfer reactions, resulting in conversions higher than 95% within 12 h and the formation of the corresponding copolymers with higher molecular weight compared to the GPE homopolymer. By the curing reaction of the multifunctional novolak glycidylether with different amounts of DM-CC, insoluble networks with gel contents of 99% were formed. The polymerization shrinkage of 3.9 vol.% for the pure novolak resin was reduced to 1.7 vol.% by the addition of 50 mol% of DM-CC. The copolymer networks showed T_g_ regions at temperatures between 20 and 120 °C.

A patent of the company 3M describes the synthesis of multifunctional CCs by the reaction of glycerol carbonate with poly(isocyanate)s (Figure 21B) [106]. The CCs were used to reduce the shrinkage of numerous epoxy-amine resins. Exemplarily, the DGEBA-based epoxy resin Epon 828 exhibited shrinkage of 4.11 vol.% during curing with isophorone diamine at 70 °C. By the addition of 10, 25 and 50 mol% of a trifunctional CC, volumetric changes in the range of −0.2 to +1.73 vol.% were observed during curing under the same conditions.

### 4.5. Other Oxygen-Containing Oligocyclic Monomers 

**Figure 22:**
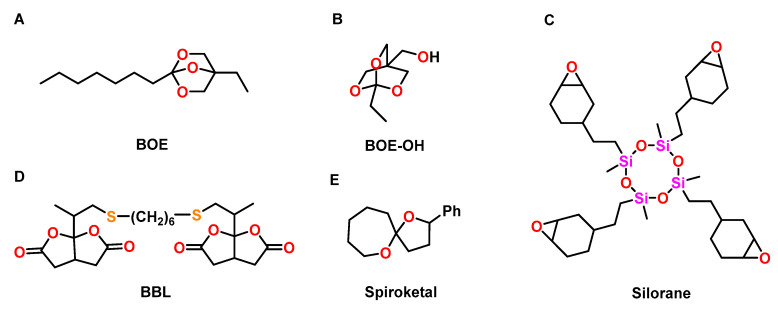
(**A**–**E**): Chemical structure of oxygen-containing oligocycles that show reduced shrinkage or even expansion during polymerization.

A bicyclic orthoester BOE (Figure 22A) was used to control the shrinkage of trimethylolpropane triglycidyl ether during cationic photopolymerization [107]. By variation of the BOE content from 0 to 50 wt.%, the formulations showed volumetric changes ranging from −3.3 to +1.6 vol.% during UV-induced cationic curing. The volumetric changes were proportional to the content of the BOE. The T_g_ of the polymer networks decreased from 110 to 40 °C with increasing amounts of the BOE. Due to flexibilization of the polymer networks, the final conversion of epoxy groups increased upon the addition of the BOE.

The thermally induced cationic copolymerization of hydroxy-modified BOE-OH (Figure 22B) with GPE was investigated by Endo and coworkers [108]. Polymerization at 130 °C in the presence of BF_3_**·**OEt_2_ as cationic initiator yielded linear, soluble polyethers. By curing at 250 °C, insoluble polymer networks were obtained due to the additional condensation reaction of hydroxy groups of the polymer chains, yielding ether bonds. During the formation of linear (co)polymers, pure GPE showed shrinkage of 7 vol.%, which was reduced to 4 vol.% upon the addition of 90 mol% of the BOE. During crosslinking at 250 °C, only slight shrinkage reduction of 0.9 vol.% was achieved. The linear polymers and the crosslinked networks exhibited T_g_s in the range of −12 to +20 °C and −22 to +47 °C, respectively.

The anionic copolymerization of epoxides with various bicyclic bis(*γ*-butyrolactone)s was investigated by the group of Endo [109]. The ring-opening polymerizations yielding polyesters were performed in THF at 120 °C using triphenylphosphine PPh_3_ as initiator. By copolymerization of a mixture of DGEBA and bifunctional BBL (Figure 22C) in the molar ratio of 50:50, polymer networks with a T_g_ of 68 °C and high thermal stability were obtained; volumetric expansion of +1.0 vol.% was observed.

Cationic double ROP of a spiroketal was investigated by Endo et al. (Figure 22D) [110]. The polymerization was performed with 5 mol% of BF_3_**·**OEt_2_, methyl triflate TfOMe, trifluoromethanesulfonic acid TfOH, or SnCl_4_ as cationic initiator in CH_2_Cl_2_ at temperatures between 50 and 100 °C. The double ROP yielded poly(ether ketone)s with M_n_s in the range from 2.9 to 3.7 kDa. Depending on the reaction conditions, the spiroketal induced volumetric expansion between +0.9 to +1.2 vol.% in the course of the polymerization.

Wienmann et al. compared the commercially available silorane (Figure 22E) with conventionally applied methacrylate-based dental composites [111]. Cationic crosslinking of the silorane composite was performed by irradiation with visible light, using an initiator system composed of camphorquinone, an iodonium salt, and a tertiary amine. The silorane composite showed shrinkage of 0.94%, which was lower compared to the methacrylic composites (shrinkage in the range of 2.0 to 3.6 vol.%). The cured silorane composites showed similar mechanical properties like the methacrylic systems. 

Lee et al. reported volumetric expansion during the cationic curing of DGEBA in the presence of *N*-benzylpyrazinium hexafluoroantimonate as initiator [112]. The density of the formed polymer networks was measured at room temperature after curing at 150 °C for (maximum) 40 min, and was monitored as a function of the curing time. While the density of the samples containing 0.5 wt.% of the initiator did not change during curing, the density of the samples containing 3.0 wt.% of the initiator decreased from 1.23 to 1.20 g·cm^−^^3^ with proceeding reaction time. This corresponds to a volumetric expansion of 2.5 vol.%. The authors explained the volumetric expansion as a consequence of inter- and intramolecular hydrogen bonding due to an increased abundance of hydroxy groups at higher initiator concentrations and the resulting changes of molecular packing in the polymer networks.

## 5. Expanding Benzoxazines

**Figure 23:**
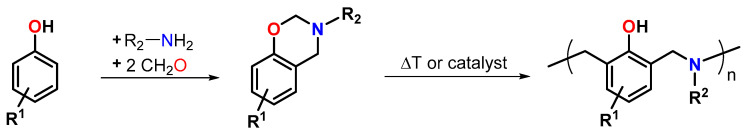
Schematic representation of the synthesis and polymerization of benzoxazine monomers.

In the last decades, benzoaxazine resins have gained increased attention as alternative to resol- and novolak-type phenolic resins [113,114]. Benzoxazine-based thermosets show high T_g_s, excellent flame retardance, high mechanical strength, low dielectric constants as well as low coefficients of thermal expansion and, hence, are used for various industrial applications such as aerospace composites and laminates for printed circuit boards PCBs [115]. Compared to classic phenolic resins, benzoxazines polymerize with low volumetric shrinkage or even slight expansion [116] and without the formation of any by-products. Typically, benzoxazines are synthesized by a Mannich-type condensation reaction from phenol derivatives, formaldehyde and primary amines (Figure 23). The ring-opening polymerization of benzoxazines proceeds at elevated temperatures (typically 140 to 220 °C) without a catalyst by cleavage of the C-O-bonds and the formation of reactive iminium ions. The addition of cationic initiators such as Lewis acids [117], Bronsted acids [118], and Crivello salts [119] accelerates the polymerization and enables curing at lowered temperatures. 

**Figure 24:**
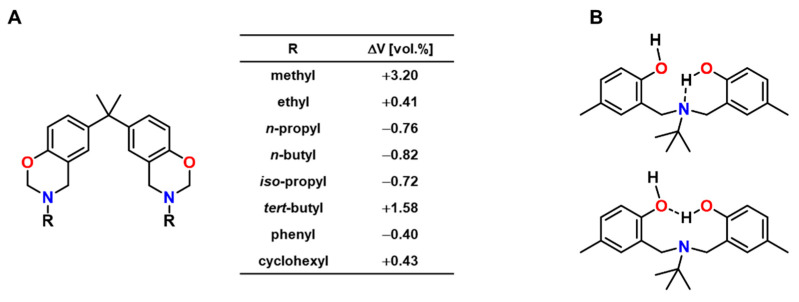
(**A**): Volumetric changes of bisphenol A-based benzoxazines with different substituents R at the nitrogen atom. (**B**): Schematic representation of possible hydrogen intramolecular bonds in *N*,*N*-bis(3,5-dimethyl-2-hydroxybenzyl)methylamine as model compound for benzoxazine-based resins.

Ishida and Low published a study of the volumetric expansion of benzoxazine resins, which were synthesized from bisphenol A, formaldehyde and various primary amines (Figure 24A) [116]. Curing at 150 °C without a catalyst or initiator yielded polymer networks with T_g_s in the range from 135 to 180 °C. The volumetric changes upon crosslinking were determined by density measurements of the monomers and the cured polymers at room temperature and were found to correlate with the substituent R of the amine employed in the monomer synthesis. For aliphatic substituents, the volumetric expansion decreased with the length of the aliphatic chain: While the methyl-substituted monomer showed expansion of +3.20 vol.%, the *n*-butyl-substituted monomer polymerized with shrinkage of 0.82 vol.%.

The authors proposed that the volumetric changes are a consequence of intramolecular hydrogen bonding and the resulting molecular packing of the polymer networks. Intramolecular hydrogen bonds between hydroxyl groups and the nitrogen bridges or between adjacent hydroxyl groups were argued to occur in benzoxazine-based resins and verified on the example of *N*,*N*-bis(3,5-dimethyl-2-hydroxybenzyl)methylamine as model dimer (Figure 24B) [116,120]. These hydrogen bonds can induce the formation of curled structures, the presence of which hinders tight packing of the polymer chains. Correspondingly, stronger hydrogen bonding results in increased expansion, while the strength of the hydrogen bonds was found to depend on the substituent R at the nitrogen atom.

**Figure 25:**
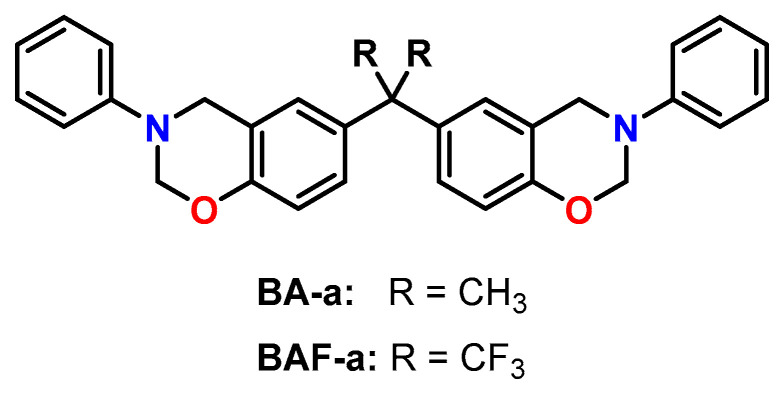
Chemical structures of the benzoxazine monomers BA-a and BAF-a.

The effect of different catalysts on the volumetric expansion of the benzoxazine BA-a was investigated by Liu et al. (Figure 25) [121]. Four different benzoxazine systems were cured at 160 °C: BA-a without catalyst, BA-a with oxalic acid, BA-a with *N,N*-dimethyl benzylamine as well as BA-a with 10 wt.% epoxy resin E44 and *N,N*-dimethyl benzylamine. Density measurements of the monomers and the cured polymer networks revealed expansions in the range from +1.19 to +1.48 vol.%. However, by following the volumetric changes during isothermal curing at 160 °C with a silicon oil-based dilatometer, shrinkage was observed for all formulations. While the pure benzoxazine exhibited curing shrinkage of 4.23 vol.%, the presence of the catalysts oxalic acid or *N,N*-dimethyl benzylamine leads to slightly reduced curing shrinkage of 3.53 and 4.10 vol.%, respectively. In addition, the presence of the catalysts accelerated the crosslinking reaction, resulting in shorter gelation times.

Lofti et al. investigated the mechanical properties and biocompatibility of poly(benzoxazine)-based composites reinforced with zirconia ZrO_2_ micro- and nanoparticles [122]. The composites were prepared by the curing of homogenized mixtures of BA-a (Figure 25) and different amounts of ZrO_2_ at 220 °C without a catalyst. Simulation of the volumetric changes during polymerization with the Materials Studio software revealed curing shrinkage of 2.8 vol.%. According to nanoindentation measurements, the elastic modulus and the hardness increased upon the addition of ZrO_2_ particles compared to the unfilled poly(benzoxazine). The biocompatibility of the composites was tested by an MTT assay with MG63 cells and revealed high cell viabilities of 97%.

The effect of curing conditions on the density and the T_g_s of poly(benzoxazine)s derived from the bisphenol A-based monomer BA-a and its fluorinated analogue BAF-a was investigated by Ishida and coworkers (Figure 25) [123]. Isothermal curing was performed without a catalyst in temperatures in ranging from 135 to 185 °C. During polymerization, both monomers showed expansions of 1–2 vol.%, while the expansion increased with the degree of conversion. At a curing temperature of 185 °C, nearly complete conversion and maximum T_g_s of 170 °C for BA-a and 220 °C for BAF-a were reached. In addition, poly(BAF-a) exhibits higher temperature stability than poly(BA-a).

**Figure 26:**
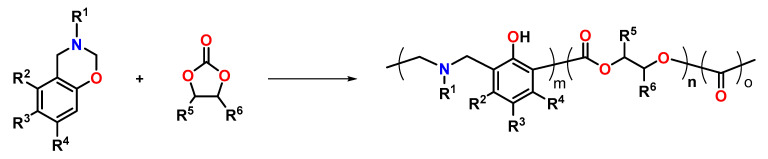
Schematic representation of the ring-opening copolymerization of benzoxazines and CCs.

Wiesbrock and Windberger described the thermally initiated copolymerization of benzoxazines with CCs upon volumetric expansion (Figure 26) [124]. The homopolymerization of the benzoxazine monomers occurred with slight expansion in the range of 1 to 3 vol.%. During copolymerization with cyclic carbonates such as ethylene and propylene carbonate, unexpectedly high expansion rates of up to 30 vol.% were observed. Highest expansion rates were observed if the molar ratio of the benzoxazines and cyclic carbonates was n:n = 1:1. The mechanical properties of the obtained poly(benzoxazine)-*co*-poly(carbonates) can be adjusted from highly brittle to rubber-like materials by variation of the carbonate content. Benzoxazine monomers that contain olefinic groups enabled the preparation of pre-cured polymer networks by UV-induced by thiol-ene click reaction at room temperature prior to the ring-opening copolymerization with the CCs. 

Saiev et al. developed a model for the calculation of the thermomechanical properties of benzoxazine thermosets [125]. Three monomers, namely 4,4′-(*p*-phenylene)-bis(3,4-dihydro-6-ethyl-2H-1,3-benzoxazine), 4,4′-(*p*-phenylene)bis(3,4-dihydro-2H-1,3-benzoxazine) and bis(3,4-dihydro-6-phenyl-2H-1,3-benzoxazinyl)isopropane were used for the simulations in this study. Molecular dynamic simulations were performed based on a newly developed crosslinking algorithm for thermal curing and enabled to monitor the evolution of the molecular structure during the polymerization reaction. Physiochemical and thermomechanical properties such as molecular weight, crosslinking density, coefficient of thermal expansion, T_g_, Young modulus and shear modulus were calculated and correlated with the network topology of the polymers. According to density calculations, the volumetric expansion during polymerization increased with the degree of conversion, e.g., volumetric expansion of +2.5 vol.% at 80% conversion.

## 6. Expanding Thiocycles

**Figure 27:**
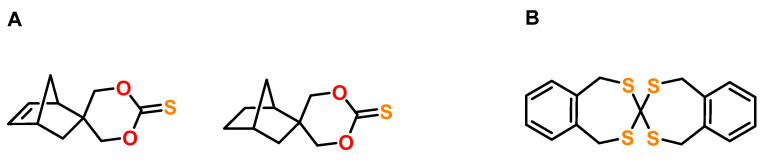
(**A**): Chemical structure of thiocarbonates. (**B**): Chemical structure of a tetrathio-SOC.

The anionic polymerization of six-membered cyclic thiocarbonates bearing norbornene and norbornane groups was investigated by Endo and coworkers (Figure 27A) [126]. The polymerizations were performed in bulk or in solutions of *N*,*N*-dimethylformamide DMF and toluene in the presence of DBU as initiator at 120 °C. Polymers with M_n_s in the range from 7.1 to 15.2 kDa were obtained with relatively low yields between 22 to 38%. For both monomers, similar expansion rates of 12.3 and 12.6 vol.% were observed. The T_g_ of both poly(thiocarbonate)s was 82 °C.

The same authors reported the cationic polymerization of a five-membered cyclic thiocarbonate bearing an adamantyl group [127]. The polymerizations were carried out in CH_2_Cl_2_ at 30 °C with 2 mol% of TfOH, TfOMe, triethyloxonium tetrafluoroborate Et_3_OBF_4_, or BF_3_·OEt_2_ as cationic initiator. During polymerization in the presence of BF_3_∙OEt_2_, volumetric expansion of 14 vol.% was observed, and polymers with a M_n_ of 10.6 kDa and Đ of 1.44 were obtained.

A tetrathio-SOC with seven-membered rings showed volumetric expansion of 5.2 vol.% during polymerization at 150 °C in the presence of tetramethylene sulfonium hexafluoroantimonate (Figure 27B) [13].

## 7. Applicability of Expanding Monomers in Novel Products and Materials

The common structural motif of the prominent classes of expanding monomers presented in this review is the presence of cyclic and even oligocyclic units. With the exception of benzoxazines, oxetanes, cyclic carbonates, and (selected) vinylcyclopropanes, the expanding monomers are not commercially available on economic price scale. Due to the limited availability of expanding monomers, strategies to enable their industrial applicability are expected to focus on the commercially available monomers and/or high-end applications with comparably low material consumption such as dental fillings (see the examples hereinabove). This shortcoming of materials’ availability, unfortunately, does not meet the plethora of diverse application fields, in which geometric accuracy of fit is highly favored in order to avoid the formation of microcracks, microvoids, internal mechanical stresses or even delamination.

On the other hand, due to the intentional volumetric expansion, comparably low T_g_s and E modulus are commonly observed in (co-)polymers obtained from expanding monomers. One straight-forward strategy to overcome the decreased thermomechanical properties is the usage of bifunctional monomers that, on the one hand, exhibit shrinkage in the course of radical-induced polymerization or crosslinking and, on the other, cationic-induced expansion due to ring-opening reactions. In particular the vinylcyclopropanes, norbornenes with cyclic carbonate groups as well as olefinic SOEs, SOCs, CCs, and benzoxazines reported hereinabove belong to this class of bifunctional monomers. Depending on the ratio of radical-induced polymerization or crosslinking and cationic-induced ring-opening, shrinkage, volume-neutral curing, or expansion can be observed. Notably, the extent can of radical-induced polymerization or crosslinking can be increased by copolymerization with other olefinic monomers that do not bear additional groups that can undergo cationic ring-opening.

Notably, in this context, bifunctional monomers, of which one of the two functionalities can be cured upon volumetric expansion, have been evaluated for applicability in novel product generations most recently. Prominent research foci comprise photopolymerizations with low or no volumetric shrinkage during curing [36,50,53,58,59,60,90,91,92,93,94,95,128,129], dental fillings with low or no volumetric shrinkage during curing [21,75,98,100,101,102,111] and the 3D printing with unrivaled alignment of the geometry of the printed object with the CAD drawing [51,73].

## 8. Summary 

Several cyclic monomers, such as cycloalkanes and cycloalkenes, oxacycles, benzoxazines, as well as thiocyclic compounds are so-called expanding monomers due to the fact that they show volumetric expansion during ring-opening polymerizations, in contrast to the vast majority of other monomers that exhibit volumetric shrinkage, which can result in the formation of microcracks, microvoids, and delamination of the polymer-based materials from adjacent surfaces. In the most recent times, in particular spiroorthoesters, spiroorthocarbonates, cyclic carbonates and benzoxazines have attracted scientific attention as expanding monomers. 

Volumetric shrinkage originates from the shortening of the Van der Waals equilibrium distance of two molecules to the length of a covalent bond. The volumetric expansion of expanding monomers (or, alternatively, reduced volumetric shrinkage) is due to the opening of rings with dense atomic packing in the course of the curing reaction. Due to the less dense atomic packing in linear structures (compared to cyclic structures), a higher degree of segmental flexibility is likely to occur in the corresponding (co-)polymers, which, correspondingly, often exhibit decreased E modulus and glass-transition temperatures. Poly(benzoxazine)s with a comparably rigid polymer structure and comparably high glass-transition temperatures are prominent counterexamples of this phenomenon.

Based on the current state-of-the-art knowledge compiled in this review, the three future trends of the research area of expanding monomers listed hereinafter are prognosed: (i) Focus on the thermomechanical properties (e.g., glass transition temperature, E modulus) of polymers and composite materials (eventually with high filling degrees); (ii) Correlation of the polymerization stimulus (thermally triggered vs. UV induced) with the material properties; (iii) Detailed screening of initiators and activators, enabling the parallel as well the sequential occurrence of crosslinking and ring-opening reactions (two-step vs. dual-stage curing mechanisms).

## Figures and Tables

**Table 1:** Methods for the quantification of volumetric shrinkage and their potential shortcomings.

Method	Potential Shortcomings
Capillary Dilatometry	Sticking of the resin to the capillary walls and the challenges of temperature control in case of strongly exothermic polymerization reactions can affect the accuracy of the measurements.
Gas Pycnometry	For the quantification of volumetric changes during curing reactions, the volume of the sample needs to be measured before and after curing.
Buoyancy Method	While the method is independent of the size or geometry of the specimens, voids inside the material and air bubbles on the surface can strongly affect the results of the measurements.
Linometry	Three-dimensional volumetric expansion can be only estimated in case of isotropic expansion.
Bonded Disk Method	The obtained values for linear shrinkage depend on the dimension of the tested specimens
Laser-Based Methods	High equipment expenditure and high security demands.
Rheometry	Three-dimensional volumetric expansion can be only estimated in case of isotropic expansion.
Thermomechanical Analyses	Standard set-ups are exclusively applicable for solid samples.
Imaging Methods	High equipment expenditure (and eventually high security demands).

**Table 2:** Boiling point, density ρ and dipole moment μ of some cyclic and acyclic carbonates.

Cyclic Carbonate	Boiling Point (°C)	ρ (g/mL)	μ (Debye)
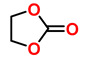	243–244	1.321	4.87
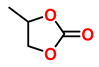	242	1.189	4.94
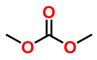	90	1.069	_
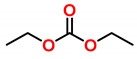	126	0.975	0.90

## Data Availability

Not applicable.

## Abbreviations

^1^H-NMR	proton nuclear magnetic resonance
AIBN	azobisisobutyronitrile
BF_3_·NEt_3_	boron trifluoride triethylamine
BF_3_·OEt_2_	boron trifluoride diethyl etherate
bis-GMA	bisphenol A glyceroate dimethacrylate
BOE	bicyclic orthoester
CAD	computer-aided design
CC	cyclic carbonate
CH_2_Cl_2_	dichloromethane
CROP	cationic ring-opening polymerization
Đ	dispersity index
DBU	1,8-diazabicyclo[5.4.0]undec-7-en
DFT	density-functional theory
DGEBA	bisphenol A diglycidyl ether
DIC	digital image correlation
DMF	*N*,*N*-dimethylformamide
EPOX	3-ethyl-3-phenoxymethyl oxetane
Et_3_OBF_4_	triethyloxonium tetrafluoroborate
FT-IR	Fourier-transformed infrared
GPE	phenyl glycidyl ether
La(OTf)_3_	lanthanide triflate
LOI	limiting oxygen index
LS	linear shrinkage
MEK	methyl ethyl ketone
M_n_	number-average molecular weight
M_w_	weight-average molecular weight
PDAC	9-phenyl-9,10-dihydroanthracen-10-ylium cation
PPh_3_	triphenylphosphine
PSOE	2-phenoxymethyl-1,4,6-trioxa-spiro[4.6]undecane
ROMP	ring-opening metathesis polymerization
ROP	ring-opening polymerization
RROP	radical ring-opening polymerization
Sc(OTf)_3_	scandium triflate
SOC	spiroorthocarbonate
SOE	spiroorthoester
TEGDMA	tri(ethylene glycol) dimethacrylate
Tetra-SOCs	tetrafunctional SOCs
TfOH	trifluoromethanesulfonic acid
TfOMe	methyl triflate
T_g_	glass-transition temperature
THF	tetrahydrofurane
TMA	thermomechanical analysis
SnCl_4_	tin(IV) chloride
UDMA	urethane dimethacrylate
UV	ultraviolet
VCP-adamantyl	1,1-bis[(1-adamantyloxy)carbonyl]-2-vinylcyclopropane
VCP-Ph	1,1-bis(phenoxycarbonyl)-2-vinylcyclopropane
VS	volumetric shrinkage
Yb(OTf)_3_	ytterbium triflate
ZrO_2_	zirconia
